# Supplementary material for: Effects of Pore-Scale Disorder on Fluid Displacement in Partially-Wettable Porous Media
Source: Sci Rep. 2016 Oct 26;6:36221. doi: 10.1038/srep36221 (PMC5080577; doi:10.1038/srep36221)
Supplement: Supplementary Information [file srep36221-s5.pdf]

Supplementary Material for: Effects of Pore-Scale Disorder on Fluid Displacement in Partially-Wettable Porous Media

Ran Holtzman[1,*]

[1]Department of Soil and Water Sciences, The Hebrew University of Jerusalem, Rehovot, Israel.
*holtzman.ran@mail.huji.ac.il

Details of the numerical model

Here we describe the essential details of the model of Holtzman and Segre[1], which was used in the numerical simulations. Our numerical model provides a mechanistic description of invasion dynamics, including capillary and viscous forces, as well as wettability effects. This is achieved by combining two complementary pore-scale modeling approaches. The first, termed "pore-based" or "dynamic pore network modeling", resolves pore pressures and interpore fluxes from pore topology and geometry[2]). The second, "grain-based", incorporates the shape of the solid particles to compute the meniscus curvature and stability[3]. In particular, it allows a mechanistic description of wettability effects in the form of cooperative pore filling, which dominates wetting invasion[3]. Combining these two approaches allows us to simulate a wide range of properties and conditions, including flow rates, wettability (contact angle) and viscosities.

Our model captures, in a highly efficient manner, the temporal and spatial nonlocality associated with rapid capillary jumps and their effect on other parts of the interface due to the much slower viscous pressure diffusion[4,5]. This provides the crucial effects of pressure screening[6] and interface readjustments[4,7]. Screening enhances the advancement of the tips of the most advanced finger relative to the "gulfs" between the fingers, prolonging the high defending fluid pressures in these gulfs[6]. Interface readjustments are caused by rapid redistribution of the defending fluid along the invasion front together with flow of invading fluid from nearby interfacial sites, reducing the capillary pressure and causing the meniscus to recede as the local curvature decreases. Readjustments lead to the disparate timescales for pore filling and bulk flow, limiting the number of pores invaded simultaneously (avalanches) by suppressing further invasion until the excess pressure in the defending fluid is dissipated by flow[4,7].

In our model, a destabilized meniscus incipiently invades the downstream pore. We evaluate the meniscus advancement and pore filling rate from the local gradient of pore pressure and the viscous resistance of each fluid. Pore pressures p are provided by enforcing the conservation of fluid mass in each pore, $\sum_j q_j = 0$ (summing fluxes q from all neighboring pores j), for a network of (1) contiguous pores occupied by same fluid and (2) pairs of pores across all unstable, advancing menisci. In other words, the front include all menisci connecting particle pairs ("throats") separating fully-invaded pores ($\Phi = 1$) from accessible, non-invaded ($\Phi = 0$) or partially-filled ($0 < \Phi < 1$) pores, where Φ is the filling status. Accessibility is determined from the topological connection with the outer boundary, so that trapped inclusions of defending fluid can form and persist.

The volumetric flow rate into a pore from its neighbor j is evaluated by assuming Stokes flow, $q_j = C_j \nabla p_j$. The interpore conductance $C \sim \rho^4/\mu_{\text{eff}}$ is evaluated from the connecting throat aperture, ρ and filling status of the invaded pore (downstream of unstable menisci), Φ. An effective viscosity, $\mu_{\text{eff}} = (\mu_i - \mu_d)\Phi + \mu_d$ allows using q to evaluate both flow of a single fluid between two pores and filling rate. Here μ_d and μ_i are the defending and invading fluid viscosities. The gradient $\nabla p_j = (p_j - p)/\Delta x_j$ is evaluated from the pressure difference between the two pores (the capillary pressure if they contain different fluids), assuming that most of the resistance occurs in the pore constriction, over a distance $\Delta x_j = \rho_j$. Interface readjustments are captured by re-emptying of partially-filled pores upon reversal of the meniscus advancement direction, $p > p_j$ and $q_j < 0$.

Computationally, we use a staggered, adaptive Euler time-stepping to capture invasion dynamics, where at each time step we (1) identify the flow network–front position and conductance C, from the filling status Φ; (2) evaluate pore pressures p; (3) check for new instabilities, update the network; (4) evaluate interpore fluxes q; and (5) update filling of invaded pores by $\Phi(t + \Delta t) = \Phi(t) + q^{\text{inv}}(t)\Delta t/V$, and advance in time, returning to (1). Here $q^{\text{inv}} = \sum_u q_u$ is the filling rate (summing over all throats with unstable menisci). The timestep Δt is chosen adaptively so that only a fraction a pore volume is filled. When pore invasion ends ($\Phi = 1$), the new interface configuration is resolved by replacing the unstable arcs with new ones that touch the upstream particle. The above provides a simple description of the invasion dynamics without explicit geometrical calculations of changes in fluid volume from changes in menisci curvature; this allows us to capture the disparate timescales of pore filling and bulk flow[4,5], a long-standing computational challenge.

Simulation conditions and parameters

We vary three control parameters: disorder (λ), flow rate (Ca) and wettability (θ). We enforce $\mathrm{Ca} = \mu_d v/\gamma$ through the volumetric rate Q, evaluated from the total volume drained during the time to breakthrough, $Q = V_{tot}/t_{tot}$. The velocity is computed from $v = Q/A_{out}$, where A_{out} is the outlet cross-sectional area. We simulate fixed rate Q by setting the hydraulic resistance of the inlet region (a disk of size of several pores) to be orders of magnitude larger than elsewhere, with a fixed pressure drop between the inlet and outermost (outlet) pores; this ensures a nearly-constant rate regardless of the interface configuration. The simulations are terminated at breakthrough (once an outlet pore is invaded).

We eliminate the effects of geometrical sample details (of a specific random seed) by using the same seed to produce samples of different disorder, stretching the particle size distribution. In all simulations we used the following parameters: γ=67$\cdot 10^{-3}$ N/m, $\mu_i = 1.8 \cdot 10^{-5}$ Pa·s, $\mu_d = 1 \cdot 10^{-3}$ Pa·s, $a = 500$ μm, and $\bar{d} = 0.54a$. Qualitatively, namely for visual identification of patterns, we used large samples of L=260a (300×520 pores). For the quantitative analysis, we perform four realizations (with different seeds) for each set of conditions, Ca (ten values) and θ (two values), for three disorder degrees, a total of 12 samples and 240 simulations. To reduce computation time, we used samples of $L = 100a$ (115×200 pores). We note that in small domains finite size effects can become dominant and quantitative results would significantly change with the system size. To test that L=100a is sufficiently large, we compared the data for L=100a with a subset of the data (several λ, Ca and θ) generated using the larger samples (L=260a). Similarity of values and trends of the main pattern characteristics (e.g. A_{inter} and A_{front}) between the two sets provided us with a qualitative confirmation that our choice of L=100a is plausible.

Experimental videos showing front propagation dynamics

Time lapse images of the experiments demonstrate the impact of disorder λ on the invasion dynamics. At low withdrawal rates ($Q = 0.5$ ml/min, $\mathrm{Ca} = 2.4 \cdot 10^{-4}$), increasing λ changes the mode of invasion from radially-symmetric and continuous–sweeping most of the defending fluid and thus resulting in a compact front (Video 1, $\lambda = 0.22$) to intermittent, non symmetric–leaving multiple trapped clusters behind, leading to capillary fingering (Video 2, $\lambda = 0.52$). These transitions are caused by the direct relationship between λ and the number of bottlenecks in the form of narrow constrictions with high entry pressures. As the rates are increased (e.g. $Q = 20$ ml/min, $\mathrm{Ca} = 9.6 \cdot 10^{-3}$), viscous instabilities dominate leading to radial growth of thin, tortuous fingers (Videos 3 and 4); increasing λ diminishes the impact of the underlying lattice, leading to a transition from ordered dendritic (Video 3, $\lambda = 0.22$) to viscous fingering (Video 4, $\lambda = 0.52$).

References

1. Holtzman, R. & Segre, E. Wettability stabilizes fluid invasion into porous media via nonlocal, cooperative pore filling. *Phys. Rev. Lett.* **164501**, 1–5 (2015).

2. Joekar-Niasar, V. & Hassanizadeh, S. M. Analysis of fundamentals of two-phase flow in porous media using dynamic pore-network models: A review. *Crit. Rev. Environ. Sci. Technol.* **42**, 1895–1976 (2012).

3. Cieplak, M. & Robbins, M. O. Influence of contact angle on quasistatic fluid invasion of porous media. *Phys. Rev. B* **41**, 11508–11521 (1990).

4. Armstrong, R. T. & Berg, S. Interfacial velocities and capillary pressure gradients during haines jumps. *Phys. Rev. E* **88**, 043010 (2013).

5. Berg, S. *et al.* Real-time 3D imaging of haines jumps in porous media flow. *Proc. Natl. Acad. Sci. U.S.A.* **110**, 3755–3759 (2013).

6. Løvoll, G., Méheust, Y., Toussaint, R., Schmittbuhl, J. & Måløy, K. J. Growth activity during fingering in a porous Hele-Shaw cell. *Phys. Rev. E* **70**, 026301 (2004).

7. Xu, L., Davies, S., Schofield, A. B. & Weitz, D. A. Dynamics of drying in 3D porous media. *Phys. Rev. Lett.* **101**, 094502 (2008).
